# Phylogenetic and biochemical analysis of calsequestrin structure and association of its variants with cardiac disorders

**DOI:** 10.1038/s41598-020-75097-3

**Published:** 2020-10-22

**Authors:** Qian Wang, Tautvydas Paskevicius, Alexander Filbert, Wenying Qin, Hyeong Jin Kim, Xing-Zhen Chen, Jingfeng Tang, Joel B. Dacks, Luis B. Agellon, Marek Michalak

**Affiliations:** 1 Department of Biochemistry, University of Alberta, Edmonton, Alberta T6G 2H7 Canada; 2 Division of Infectious Disease, Department of Medicine, University of Alberta, Edmonton, AB T6G 2G3 Canada; 3 Institute of Biomedical and Pharmaceutical Sciences, Key Laboratory of Fermentation Engineering, Hubei Provincial Cooperative Innovation Center of Industrial Fermentation, Hubei Key Laboratory of Industrial Microbiology, Hubei University of Technology, Wuhan, Hubei China; 4 Department of Physiology, University of Alberta, Edmonton, Alberta T6G 2H7 Canada; 5 School of Dietetics and Human Nutrition, McGill University, Ste. Anne de Bellevue, Quebec, H9X 3V9 Canada

**Keywords:** Biochemistry, Proteins, Membrane proteins

## Abstract

Calsequestrin is among the most abundant proteins in muscle sarcoplasmic reticulum and displays a high capacity but a low affinity for Ca^2+^ binding. In mammals, calsequestrin is encoded by two genes, *CASQ1* and *CASQ2*, which are expressed almost exclusively in skeletal and cardiac muscles, respectively. Phylogenetic analysis indicates that calsequestrin is an ancient gene in metazoans, and that the duplication of the ancestral calsequestrin gene took place after the emergence of the lancelet. *CASQ2* gene variants associated with catecholaminergic polymorphic ventricular tachycardia (CPVT) in humans are positively correlated with a high degree of evolutionary conservation across all calsequestrin homologues. The mutations are distributed in diverse locations of the calsequestrin protein and impart functional diversity but remarkably manifest in a similar phenotype in humans.

## Introduction

The sarcoplasmic reticulum (SR) is a high specialized membrane network that supports mechanical muscle functions requiring large fluxes of Ca^2+^. Consequently, the SR controls excitation–contraction coupling^1–3^ without compromising Ca^2+^ requiring cellular processes that are normally associated with the endoplasmic reticulum^4^. There are two well defined structural and functional regions of the SR in cardiac muscle: the longitudinal SR that runs parallel to the myofibrils and the junctional SR that forms multiple membrane contacts with T-tubule membrane contact sites^5–7^. SR-T-tubules membrane contact sites play an important role in initiation of the excitation–contraction coupling^7^. The longitudinal SR is enriched with Ca^2+^-ATPase (SERCA) which is responsible for the rapid removal of Ca^2+^ from the cytoplasm to initiate muscle relaxation^1,5,6^. The junctional SR is the primary site of Ca^2+^ release via the Ryanodine Receptor (RyR)/Ca^2**+**^ channel which triggers muscle contraction. The SR luminal Ca^2+^ binding protein calsequestrin is a high capacity, low affinity Ca^2+^ binding protein that forms oligomeric structures that regulate RyR activity via interactions with RyR, triadin and junctin^1,8–14^. There are two isoforms of calsequestrin, which are encoded by two different genes: cardiac calsequestrin (*CASQ2*) and skeletal muscle calsequestrin (*CASQ1*)^14,15^. The crystal structure of calsequestrin indicates that the protein contains three thioredoxin-like domains reminiscent of ER luminal oxidoreductases^16,17^.

Catecholaminergic polymorphic ventricular tachycardia (CPVT) is an inherited disease characterized by ventricular arrhythmias leading to sudden death^18,19^. CPVT results from defects in intracellular Ca^2+^ handling by cardiomyocytes. Two major variants have been associated with the CPVT disorder. The autosomal dominant form is associated with mutations in the cardiac ryanodine receptor (RyR2) gene and accounts for ~ 50% cases, while a recessive form with mutations in the cardiac isoform of calsequestrin (CASQ2) accounts for 2–5% cases. Other mutations also found in the *CALM1*, *CALM2*, *CALM3* (encodes calmodulin1, 2, 3 respectively), and *TRDN* (encodes triadin) gene account for < 2% of CPVT cases^20–24^. Thirteen mutations in the CASQ2 gene have been identified in CPVT patients, in sudden death syndrome^25,26^, and three of them are non-synonymous polymorphisms (cSNP)^24,27^. Several biochemical and cell biological studies of R33Q, L167H, and D307H calsequestrin mutants indicate that these mutations lead to impaired Ca^2+^ storage and Ca^2+^ release from the SR^22,24–41^. Recently new calsequestrin mutations have been identified including K180R, D351G, G332R, and P329S^27,39,40,42–46^.

In this study, we examined the evolutionary constraints of the CPVT related calsequestrin mutations, and included *Casq1*, *Casq2* and pre-duplicate calsequestrin in the phylogenetic analysis. We showed that calsequestrin is an ancient protein in the metazoan, and that the duplication of the calsequestrin gene took place after the divergence of the lancelet but before divergence of Chondrichthyes. We noted that calsequestrin mutations, associated with CPVT, positively correlated with an increase in the degree of evolutionary conservation of the mutated sites. Furthermore, we carried out biochemical and biophysical analysis of seven CPVT related mutants (R33Q, L167H, D307H, K180R), and whole exome sequencing variants (D351G, G332R, P329S) selected based on their linked to the human CPVT phenotype and located within highly conserved thioredoxin-like domain. The mutations are distributed in diverse locations of the calsequestrin protein but remarkably manifest in a similar phenotype in humans.

## Results

### Emergence and specialization of calsequestrin within animals

**Figure 1:**
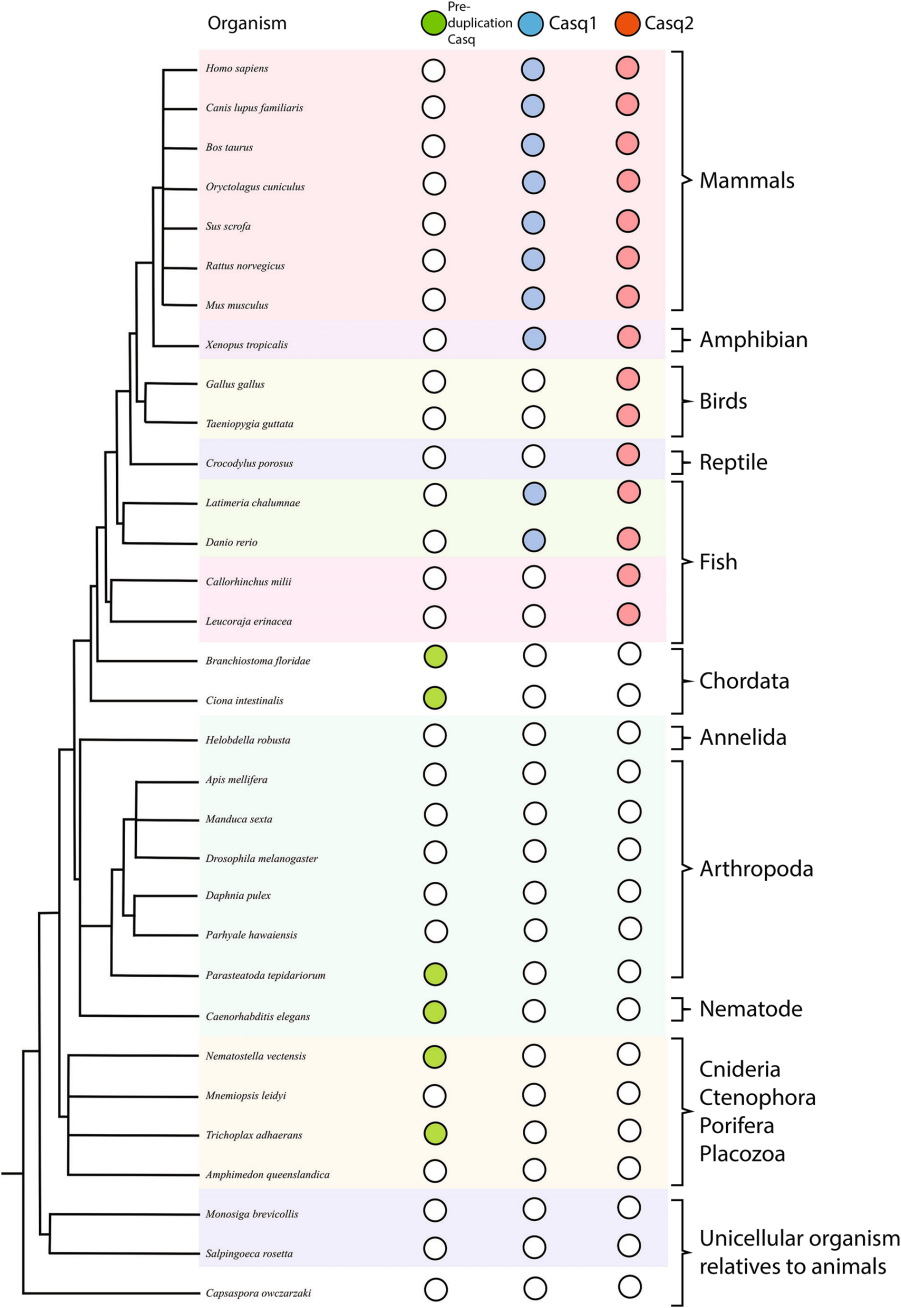
Calsequestrin homologues in the vertebrate and invertebrate lineages. Empty circles indicate the gene is absent. Green circles indicate the presence of pre-duplication Casq, blue circles the presence of *casq1*, and red circles the presence of *casq2*.

Phylogenetic analysis of the two calsequestrin genes (*casq1* and *casq2*) was carried out with the aim of clarifying the distribution and conservation of each paralogue across animals, and thus deduce the timing of the gene duplication event and relate this information to calsequestrin mutants responsible for CPVT. Homology searching was undertaken in 23 metazoan genomes and three outgroup lineages to identify calsequestrin homologues. We identified unambiguous calsequestrin homologues in most of the vertebrate and invertebrate lineages (Supp. Table S1). Furthermore, we revealed that *Ciona intestinalis*, *B. floridae,* and all taxa within the invertebrates possess a single calsequestrin gene, including taxa as deeply branched as Trichoplax and Nematostella (Fig. 1). We did not identify a calsequestrin homologue in the sponge Amphimedon despite rigorous searches. This is potentially due to a database error, but more intriguingly may well represent a loss of calsequestrin in this lineage, since preliminary searches of other sponge genomes also failed to identify calsequestrin homologues. Although the relative branching order of the basal lineages within animals is still disputed, with Nematostella or sponges as the deepest branch, calsequestrin is clearly an ancient protein within the metazoan (Suppl. Figs. S1 and S2).

Each of these lineages, as well the invertebrates and hemichordates, possess only a single calsequestrin gene, leaving the timing of when *casq1* vs c*asq2* arose as an outstanding question. Preliminary phylogeny provided moderate support for the non-vertebrate sequences emerging basal to clades of the *casq1* and *casq2*, thus being pre-duplicated versions (Suppl. Figs. S1). Further analysis focused on vertebrate gene sequences and using the lancelet sequences as an outgroup (Suppl. Fig. S2). The analysis robustly showed that the calsequestrin gene duplication that gave rise to *casq1* and *casq2* occurred after the divergence of the lancelet lineage but before the divergence of the Chondrichthyes (i.e. cartilaginous fish). We were unable to identify any calsequestrin sequences in the insect lineage. This likely represents a bona fide loss in this line given the positive identification of a homologue in the spider *Parasteatoda tepidariorum*.

We believe the Chondrichthyes and Avian taxa lost *casq1* independently. We identified *casq1* paralogues in the Chondrichthyes despite robustly classifying *casq2* being present and the duplication having taken place prior to this point (Suppl. Fig. S2). The same was observed for the avian taxa sampled, suggesting that *casq1* was lost independently in these three lineages. In mammalian muscles the two calsequestrin isoforms exhibit tissue specific expression^47–49^. Casq2 is expressed in cardiac and slow-twitch skeletal muscle, whereas Casq1 is expressed in adult fast-twitched muscle^47–49^. Cartilaginous fish as well as avian animals have both fast-twitch and slow-twitch skeletal muscles even though they appear to lack *casq1*.

### Conservation of CPVT associated Casq2 mutants throughout animal kingdom

**Figure 2:**
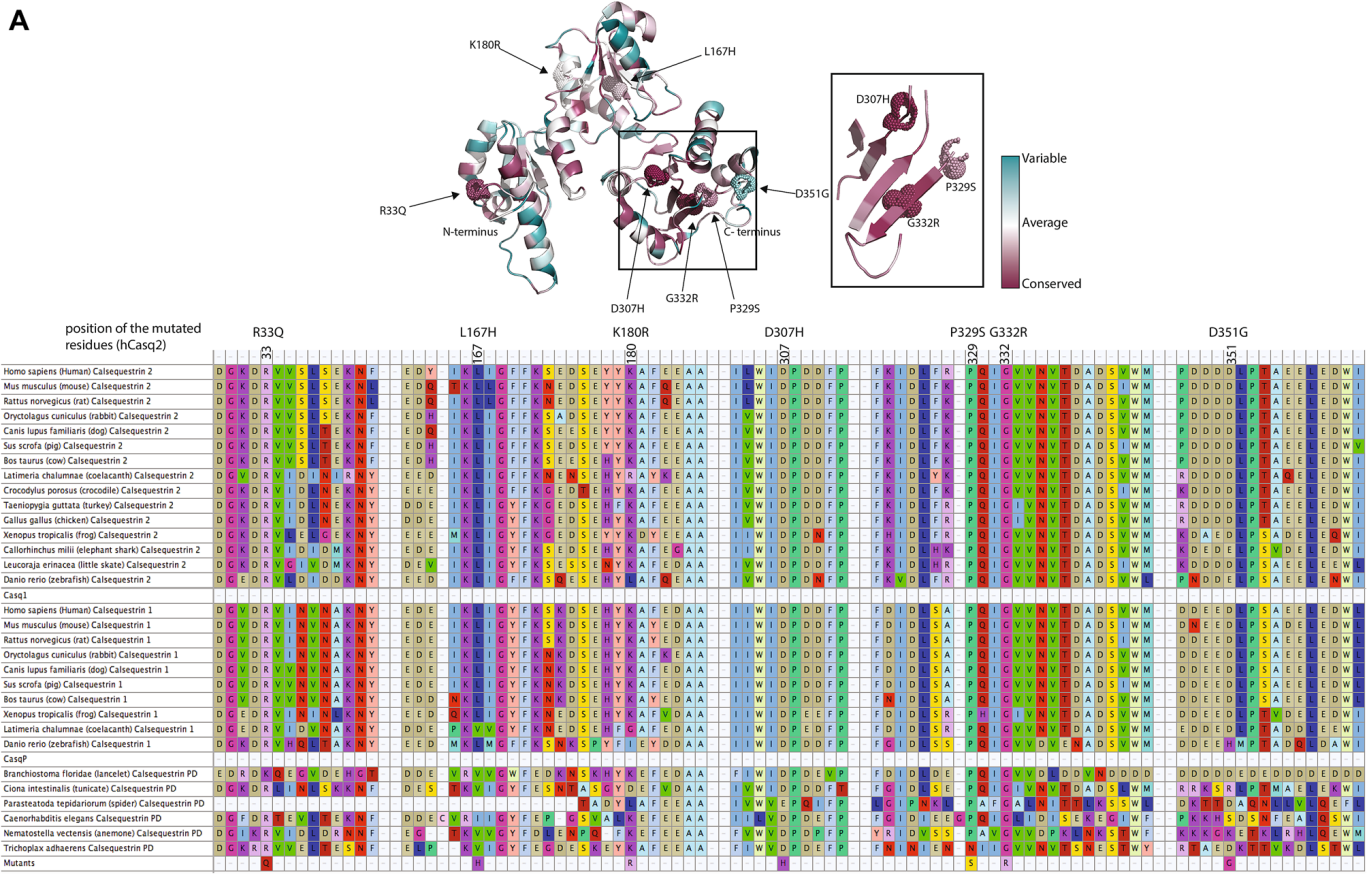

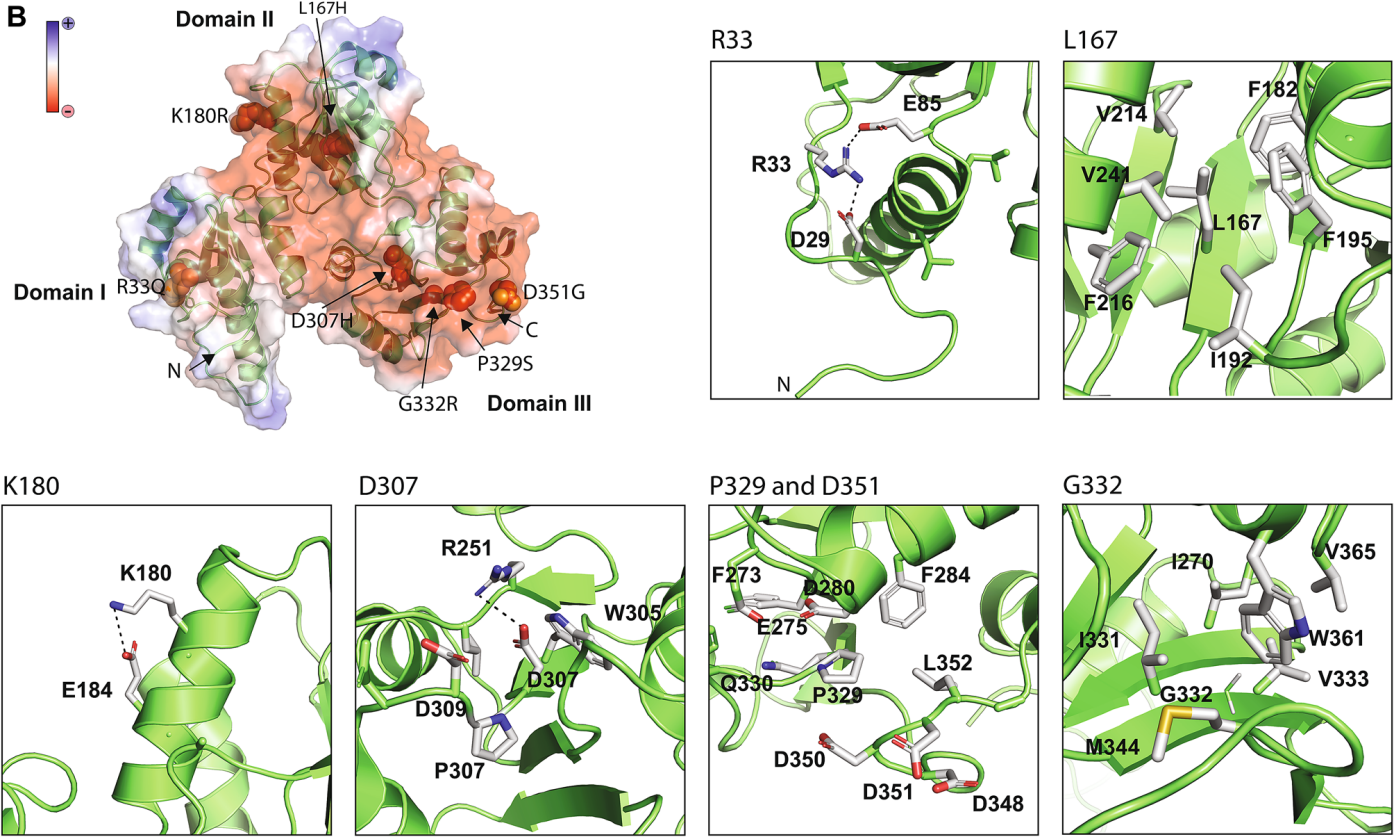
Amino acid sequence alignments and calsequestrin 3D structure. (**A**) The 3D structure of the cardiac isoform of calsequestrin (Casq2) is shown (2VAF). The location of R33Q, L167H, K180R, D307H, P329S, G332R, and D351G Casq2 mutants are depicted in the structure. A scale of variable to conserved residues is indicated in the Figure. Mutants are shown as dot sphere. The highly conserved beta-sheet from the third thioredoxin-like fold is enlarged in the box and shown separately. The table shows the alignment of calsequestrin amino acid sequences. The degree of conservation of Casq2 sequences is color-coded using ConSurf ^71^. Multiple sequence alignments were inputed from the Casq1, Casq2, and Casqp alignment. Different colors represent similar/identical amino acid residues. The location of mutated residues is indicated in the table. hCASQ2, human Casq2. (**B**) Human Casq2 (PBD: 2VAF) with electrostatic potential surfaces. The location of the N- and C-terminus of Casq2 is indicated. Secondary structures are shown as green ribbons, the amino acid residues corresponding to the CPVT related mutations are indicated by the corresponding van der Waals surface as orange spheres. Local environments around R33, L167, K180, D307, P329, G332, and D351 residues are shown. Secondary structures are shown as green ribbons, dotted lines indicate a polar interaction between side-chains. Oxygen, nitrogen, and carbon atoms are shown in red, blue, and white, respectively. For G332, the hydrogens on α-carbon are shown in white. Prepared using PyMOL v2.4.1 (https://www.pymol.org/2/).

Having the evolutionary distribution of *casq1* and *casq2* allowed us to assess the conservation of sites in Casq2 proteins where calsequestrin mutations in the human *CASQ2*, have been associated with CPVT (Fig. 2A,B)^24,50,51^. We selected the following seven Casq2 mutants for further analyses: R33Q, L167H, D307H, K180R, P329S, G332R, and D351G (Fig. 2A,B). Many mutations are scattered across the three thioredoxin-like domains of Casq2 (Fig. 2A), but remarkably they all lead to a similar clinical outcome^24,50^. L167H, and D351G are conserved in Casq1 and Casq2 paralogues found in vertebrates but are variable in the pre-duplicated non-bilaterian (Fig. 2A). In contrast, positions R33Q, K180R, D307H, P329S and G332R are fully conserved across all calsequestrin homologues including pre-duplication Casq (Suppl. Fig. S2).

### Ca^2+^ binding to Casq2 mutants

**Figure 3:**
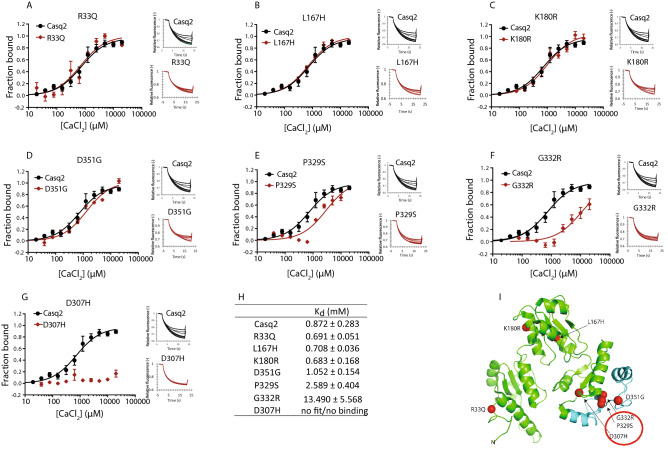
Calcium binding to calsequestrin mutants. (**A**–**G**) MST analysis of Ca^2+^ binding to mutants Casq2 (red line) and wild-type Casq2 (black line). (**H**) Calculated Ca^2+^ binding affinities of Casq2 mutants. (**I**) 3D structure of human Casq2 (adapted from 2VAF) with the location of mutants indicated in the Figure. The red circle depicts the location of mutations that affected Ca2 binding to Casq2.

With the conservation of the relevant amino acid positions in hand, we next carried out biochemical and biophysical analysis of the Casq2 mutants in order to gain insight into the contribution of these mutations to the development of CPVT. First, we used microscale thermophoresis (MST) to investigate Ca^2+^ binding to Casq2 mutants. Mutation of Casq2 residues R33Q, L167H, K180R and D351G had no significant effect on Ca^2+^ binding to calsequestrin (Fig. 3A–D) with K_d_ values ranging from 0.872 ± 0.283 mM for wild-type to 1.052 ± 0.154 mM for the D351G mutant (Fig. 3H). However, P329S and G332R mutants exhibited altered Ca^2+^ binding affinities (Fig. 3E,F). Previous studies showed that R33Q, L167H, and D307H have highly reduced Ca^2+^ binding capacity^17,31^. Here, using label-free MST assay the D307H Casq2 mutant showed no measurable Ca^2+^ binding (Fig. 3G). Of the seven mutants associated with CPVT examined, only three exhibited altered Ca^2+^ binding properties.

### Conformational changes and protein folding of Casq2 mutants

**Figure 4:**
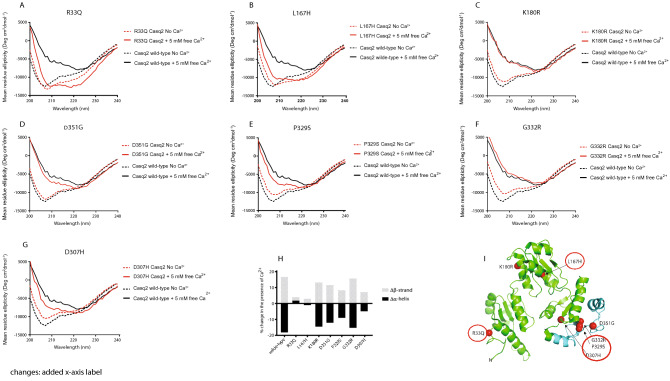
CD analysis of Casq2 mutants. (**A**–**G**) CD analysis of Casq2 mutants (red solid line) and wild-type Casq2 (black solid line). CD analysis in the presence of 5 mM Ca^2+^ is indicted by black dotted lines for wild-type Casq2 and by red dotted lines for Casq2 mutants. (**H**) Change in % of α-helix and β-strand content with the absence or presence of Ca^2+^ for wild-type Casq2 and each Casq2 mutant. Negative and positive values indicate a loss or gain of secondary structure content, respectively. (**I**) Three-dimensional structure of human Casq2 (2VAF). Red circles depict the location of mutations with altered CD spectrum.

Casq2 undergoes conformational change upon Ca^2+^ binding, and this was monitored by circular dichroism (CD) analysis^52^. Upon adding Ca^2+^, wild-type Casq2 lost 18.3% α-helix and gained 16.79% β-sheet conformation (Fig. 4)^52^. CD spectra for K180R and D351G mutants overlapped with those of the wild-type Casq2 (Fig. 4C,D,E,F), indicating no effect of the K180R and D351G mutation on the protein conformation. However, the G332R and P329S mutants, showed altered sensitivity to Ca^2+^-induced conformational changes (Fig. 4E,F). This was in agreement with the reduced Ca^2+^ affinity of these mutants (Fig. 3). In contrast, the CD spectra of mutants R33Q, L167H, and D307H revealed increased α-helix content that was not sensitive to addition of Ca^2+^ (Fig. 4A,B,G; Table 1).

**Table 1:** CD analysis of calsequestrin mutants.

Casq2	WT	R33Q	L167H	K180R	D307H	P329S	G332R	D351G
**No Ca**^**2+**^								
% α-helix	26.18	28.83	26.71	23.7	24.055	21.36	20.37	24.31
% β-sheet	19.65	17.93	19.5	21.78	20.98	22.66	23.41	21.01
**5 mM Ca**^**2+**^								
% α-helix	7.88	30.78	25.48	8.95	19.485	12.17	4.86	11.88
% β-sheet	36.44	20.05	22.56	35.17	26.96	31.05	39.13	32.51
Δ α-helix	− 18.3	1.95	− 1.23	− 14.75	− 4.57	− 9.19	− 15.51	− 12.43
Δ β-sheet	16.79	2.12	3.06	13.39	5.98	8.39	15.72	11.5

WT, wild-type.

**Figure 5:**
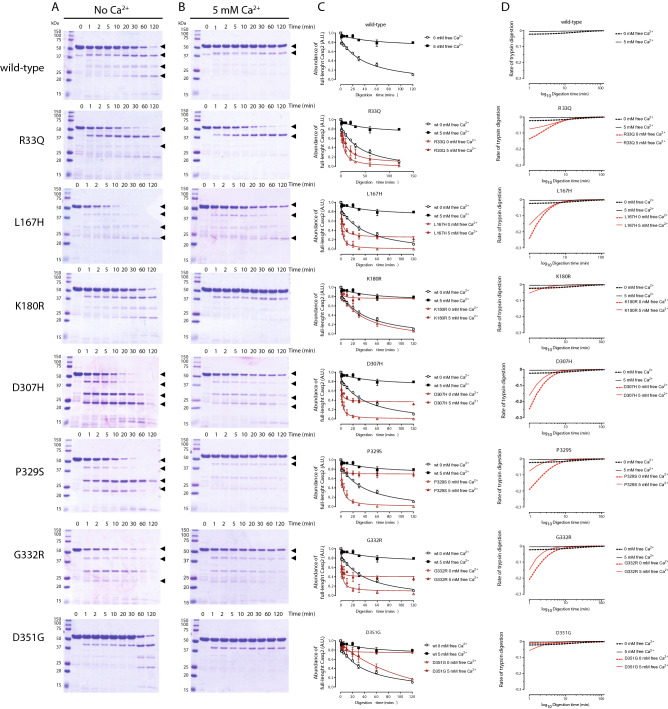
Limited trypsin digestion of Casq2 mutants. Wild-type Casq2 and Casq2 mutants were subjected to trypsin proteolysis in the absence (**A**) and presence (**B**) of 5 mM Ca^2+^ followed by SDS-PAGE. The tryptic fragments, which differed from that of wild-type Casq2 are indicated by the arrowheads. (**C**) The rate of proteolysis of wild-type and mutant Casq2 as a function of time of trypsin digestion. (**D**) The rate of proteolysis plotted as the first derivative of the fitted curve from (**C**).

Next, we tested susceptibility of the Casq2 and Casq2 mutants to trypsin digestion to further analyze the impact of Casq2 mutations on protein folding. K180R and D351G mutants showed trypsin digestion patterns similar to wild-type protein indicating no major folding differences between these proteins (Fig. 5). In support of the CD analysis, R33Q, L167H, and D307H mutants showed limited trypsin susceptibility compared to wild-type protein both in the absence and presence of Ca^2+^ (Fig. 5). R33Q, L167H, and D307H mutants showed more α-helix (Fig. 4) and an increased sensitivity to trypsin digestion in the absent of Ca^2+^ (Fig. 5), indicative of altered protein folding. In agreement with Ca^2+^ binding (Fig. 3E,F) and CD analysis (Fig. 4E,F) trypsin digestion of P329S and G332R mutants also showed increased kinetics of digestion in the presence of Ca^2+^ (Fig. 5C,D), indicative of Ca^2+^-induced conformational changes of these mutants.

**Figure 6:**
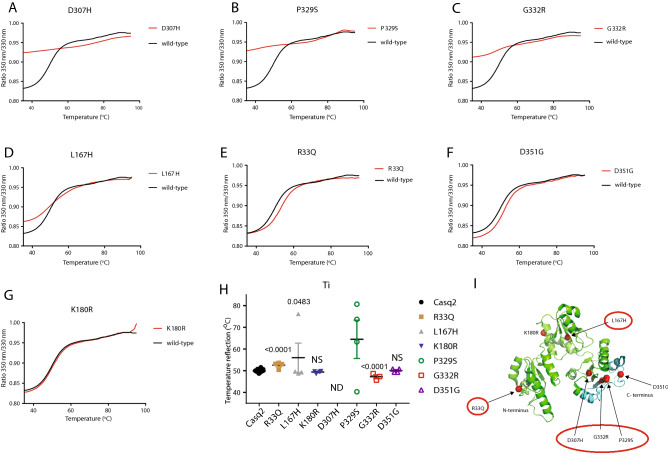
Thermal denaturation analysis of Casq2 and Casq2 mutants. (**A**–**G**) Thermal denaturation analysis of wild-type Casq2 (black lines) or Casq2 mutants (red lines) was monitored by intrinsic tryptophan fluorescence of proteins in response to increased temperature from 35 to 95 °C. Graphs represent 3 independent measurements. (**H**) Inflection temperature for Casq2 and Casq2 mutants representing the temperature at which the transition from folded protein to unfolded state occurs. (**I**) Human Casq2 crystal structure (2VAF). Mutants with significant difference in their protein folding are indicated by red circles. The location of tryptophan residues are depicted as yellow sticks.

In agreement with previous study^31^, we found D307H to be more susceptible to trypsin digestion at high Ca^2+^ concentration, and conformational changes of this mutant were less sensitive to increased Ca^2+^ concentration (Figs. 4 and 5). In absence of Ca^2+^, D307H was shown to have trypsin susceptibility and CD spectra similar to wild-type protein^17,31^. In the present study we found D307H protein was more sensitive to trypsin digestion (Fig. 5). This was further supported with altered CD spectra of the D307H mutant (Fig. 4; Table 1). The differences between the two studies is likely due to different buffer conditions used for CD analysis and trypsin digestion. As previously reported^53^, L167H and R33Q were more susceptible to trypsin digestion in absence Ca^2+^ or presence of Ca^2+^ indicating altered protein folding of the mutants. This was supported by CD analysis (Fig. 4) and thermal denaturation assays (Fig. 6; Table 2).

**Table 2:** Ti values for Casq2 and Casq2 mutants.

Calsequestrin (Casq2)	Ti value
Wild-type	49.9 ± 0.16 (n = 18)
R33Q	52.5 ± 0.73 (n = 4)
L167H	56.0 ± 6.73 (n = 4)
K180R	49.4 ± 0.29 (n = 4)
D351G	50.0 ± 0.43 (n = 4)
P329S	64.5 ± 8.82 (n = 4)
G332R	47.2 ± 0.87 (n = 3)
D307H	ND

Next, we used the Tycho NT.6 system to carry out thermal denaturation analysis of wild-type Casq2 and Casq2 mutants in a label-free environment as another indicator of protein folding. The analysis is based on measurement of the protein’s intrinsic tryptophan fluorescence and records a protein’s unfolding profile in real-time. Casq2 has 5 tryptophan residues all located in the third thioredoxin-like domain^16,17^ and fully buried in the hydrophobic core. K180R and D351G mutants showed an unfolding profile (Fig. 6A) and inflection temperature (Ti, proportionally to protein melting temperature) values (Fig. 6F,G; Table 2) similar to wild-type Casq2. Ti values for R33Q (52.55 °C) and G332R (47.23 °C) mutants, although close to the wild-type Casq2 (49.89 °C), were statistically different (Fig. 6C,E). P329S, G332R and D307H mutants showed minimal (for P329S and G332R mutants) to no detectable (for D307H mutant) unfolding transition (Fig. 6; Table 2). These mutants also showed a significantly higher initial ratio (350 nm/330 nm at 35 °C), indicating that tryptophan residues in P329S, G332R and D307H mutants were exposed to solvent, and the polarity of the local tryptophan environment was unchanged upon denaturing at higher temperature. The L167H mutant had an intermediate unfolding profile and significantly increased Ti value (Fig. 6; Table 2), indicating partially exposed tryptophan. The L167H mutation resulted in a partial disruption of the third thioredoxin-like domain and/or destabilized another protein domain, whereas, D307H, P329S, and G332R exhibited a large disruption in the third thioredoxin-like domain (Fig. 6; Table 2).

### Ca^2+^ dependent polymerization of Casq2 mutants

**Figure 7:**
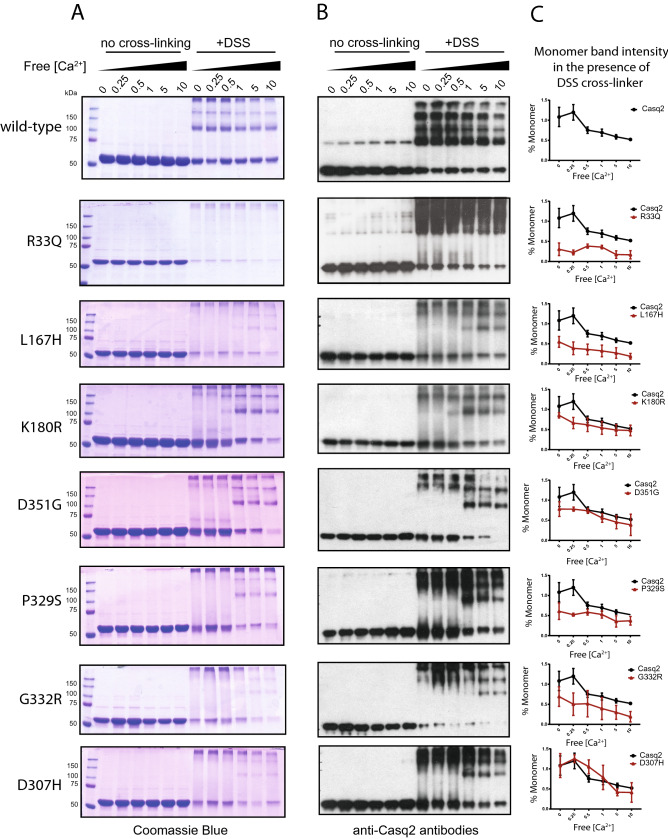
Ca^2+^-dependent polymerization of Casq2 and Casq2 mutants. (**A**) Coomassie blue stained SDS-PAGE of Casq2 and Casq2 mutant incubated with or without cross-linker at increasing free Ca^2+^ concentration. (**B**) Immunoblots were probed with anti-Casq2 antibodies. The full-length blots are shown in Figs. S4 and S5. (**C**) Quantitative analysis of Casq2 monomer (~ 50 kDa protein band) of wild-type or mutant proteins in the presence of cross-linker (from **A**) as a function of increased free Ca^2^ concentration.

**Figure 8:**
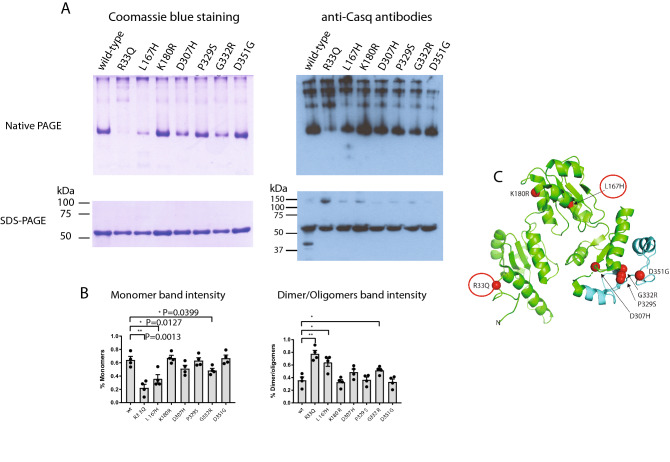
Polymerization of Casq2 mutants. (**A**) Polymerization of Casq2 or Casq2 mutants was carried out at 167 µM free Ca^2^ followed by SDS-PAGE or native gel electrophoresis. Immunoblots were probed with anit-Casq2 antibodies. A representative of four independent experiments is shown. The full-length blots are shown in Fig. S6. (**B**) Quantitative analysis of monomeric and oligomeric forms of Casq2 mutants. (**C**) Human Casq2 crystal structure (2VAF). Red circles depict the location of Casq2 mutations with highly increased oligomerization.

Casq2 undergoes monomer to oligomer transition and oligomerization^54^. Upon binding to Ca^2+^, Casq2 undergoes reversible polymerization, and this affects Casq2 assembly to the junctional SR, which could have direct impact on SR Ca^2+^ supply and RyR2 regulation^54^. We tested for a Ca^2+^-dependent oligomerization of Casq2 mutants using disuccinimidyl suberate (DSS) cross-linker (Fig. 7) and native gel electrophoresis techniques (Fig. 8). Addition of Ca^2+^ to wild-type Casq2 increased oligomerization of the protein (Fig. 7). A similar pattern of Ca^2+^-dependent oligomerization was seen for K180R, D351G and D307H mutants (Fig. 7). Surprisingly, the D307H mutant that did not bind Ca^2+^ (Fig. 3) but showed Ca^2+^-dependent oligomerization indistinguishable from the wild-type Casq2 (Fig. 7) suggesting a role of Ca^2+^ in function of this mutant. R33Q, L167H, P329S and G332R mutants had increased Ca^2+^-dependent oligomerization whereas R33Q mutant showed no dependence on Ca^2+^ for oligomerization (Fig. 7). Under conditions of native electrophoresis, wild-type Casq2 and Casq2 mutants exhibited spontaneous oligomerization (Fig. 8) with R33Q, L167H and G332R mutants having a greater proportion in oligomeric form as compared to wild-type protein (Fig. 8). This was particularly evident for the R33Q and L167H mutants which existed predominantly (> 80% and > 60%, respectively) in an oligomeric form (Fig. 8).

### Casq2 binding to IRE1α, an ER/SR stress sensor

**Figure 9:**
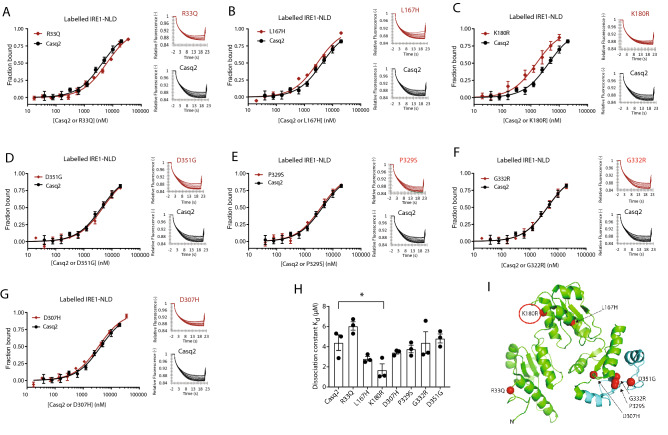
Casq2 mutants binding to the ER luminal domain of IRE1α. (**A**–**G**) Recombinant N-terminus luminal domain of IRE1α (IRE1-NLD) protein was covalently labeled with a red fluorescent tag and incubated with increasing amounts of Casq2 or Casq2 mutant as indicated in the Figure. Normalized MST time traces are shown to the right of the graph. Each data point is the average of three independent microscale thermophoresis measurements. (**H**) Bar graph depicts dissociation constants for different Casq2 mutants. *p = 0.05. (**I**) Human Casq2 crystal structure (2VAF). The red circle on the Casq2 3D structure depicts the location of mutation with altered IRE1-NLD binding.

Casq2 binds to IRE1α, an ER/SR stress sensor and squelches IRE1α activity^55^. We used MST thermophoresis to test whether Casq2 mutations affected Casq2 interaction with the luminal domain of IRE1α. R33Q, L167H, D307H, P329S, G332R and D351G bound to the luminal domain of IRE1α with similar kinetics and affinities as seen for wild-type protein (Fig. 9). However, the K180R mutant showed increased binding affinity (Fig. 9) indicating a stronger interaction between the K180R Casq2 mutant and the IRE1α luminal domain. We concluded that all Casq2 mutants tested bound to the IRE1α stress sensor.

## Discussion

Our phylogenetic analysis of the calsequestrin genes (*casq2*, *casq1*, and pre-duplication *casq*) revealed that calsequestrin is an ancient protein within the metazoan, and duplication of the calsequestrin gene took place after the divergence of the lancelet but before divergence of Chondrichthyes. Duplication of the calsequestrin gene allowed for the differentiation of a muscle-specific form of the protein, namely cardiac calsequestrin (*casq2*) expressed in cardiomyocytes. In the heart Ca^2+^ release from the calsequestrin (Casq2) rich junctional SR is initiated by the Ca^2+^-induced Ca^2+^ release mechanism. In skeletal muscle, where Casq1 is expressed, Ca^2+^ release from the junctional SR is initiated by the depolarization-induced Ca^2+^ release mechanism. Notably in mammalian species *Casq1* is almost exclusively expressed in skeletal muscle (Suppl. Fig. S3). However, *Gallus gallus* appears to have lost the Casq1 gene and *Casq2* is highly expressed in both heart and skeletal muscle tissue (Suppl. Fig. S3). Other non-mammalian vertebrates also did not show the tissue-specific expression patterns of Casq1 vs 2 observed consistently in mammalian species suggesting that this is a later evolved mammalian-specific trait.

We also discovered that the Chondrichthye and Avian taxa lost *casq1* but retained the *casq2*. Arthropoda do not have the calsequestrin gene, although they have transversely striated muscle similar to the vertebrate skeletal muscle^56^ with well-developed transverse tubules associated SR cisternae^57,58^. Considering that Arthropoda move by means of their segmental appendages, they may not require high capacity Ca^2+^ stores.

Comparison between the evolutionary distribution of calsequestrin and the RyR proteins^58^ provides insight into the evolution of the excitation–contraction coupling system in metazoan muscles. While we were unable to identify calsequestrin homologues, RyR homologues have been found in the genomes of holozoans such as Capsaspora and choanoflagellates, suggesting that the RyR protein evolved first. The pairing of the calsequestrin and ryanodine receptors seems not to have been established as essential in non-vertebrate animals, given the lack of calsequestrin in sponges but presence in cniderians and ctenophores and the opposite for the RyRs^58^. The presence of the preduplicated RyR but absence of calsequestrin in insects suggests lack of essentiality of the paired regulation as well. However, both systems have been subject to expansions likely associated with two whole-genome duplication events near evolution of vertebrates^58^. Lineage-specific losses of the families are seen here as well in both cases. For example, amphibians lack the gene encoding RyR2, yet they express both forms of calsequestrin. These results support the notion of the appearance of multiple homologues of junctional SR proteins, including calsequestrin and RyR, which are associated with depolarization-induced Ca^2+^ release or Ca^2+^-induced Ca^2+^ release mechanisms^58^.

Phylogenetic analysis of *casq2* within the metazoan revealed a high level of conservation, especially in the beta-sheet in the hydrophobic core of the third thioredoxin-like fold. The C-terminal Asp rich domain of Casq2, responsible for high capacity low affinity Ca^2+^ binding, remained highly conserved throughout many different species. Of note, the pre-duplicated calsequestrin C-terminal domain, however, contains limited numbers of acidic residues, indicating a relatively low Ca^2+^ binding capacity in this basal lineage^59^. There are many highly conserved amino acid residues distributed throughout Casq2 that may be under evolutionary constraints, and mutations in these regions of the protein are expected to impact protein structure and function^60,61^. Not surprisingly, Casq2 mutations associated with CPVT are dispersed throughout different protein regions, but all are highly conserved throughout metazoans some (R33Q, K180R, D307H, P329S and G332R) even including pre-duplication Casq. Because of a specific disease phenotype of Casq2 mutants, one a priori prediction is that the mutations associated with CPVT would be in sites conserved in Casq2 but divergent in Casq1 and preduplicates, providing gain-of function amino acid changes in Casq2 which were then disrupted by the disease phenotype-associated mutations. However, this was not what we observed. Instead we found strong conservation at these positions between the paralogues or indeed across all calsequestrin homologues. This suggests that the residues at these positions are critical for calsequestrin function. Specificity of cardiac disease seen with Casq2 mutants is likely due to the tissue-specific expression patterns of the paralogues. In humans there is little or no Casq1 paralogue expressed in cardiac tissue to compensate for Casq2 malfunction in CPVT.

For our biochemical studies, we have selected mutants linked to the human CPVT phenotype and located within highly conserved positions, namely, R33Q, L167H, K180R, D307H and three *CASQ2* variants from whole-exome sequencing clinical testing, P329S, G332R, and D351G^24–27,39,46,62,63^. In agreement with previous reports^25,30,34,36,53,64^, our studies showed that Casq2^R33Q^, Casq2^L167H^, and Casq2^D307H^ differed in their biochemical properties. Casq2^D307H^ was reported to have significantly reduced Ca^2+^ binding capacity^17^. In this study, in label-free MST Ca^2+^ binding assay the D307H mutant showed no measurable Ca^2+^ binding and altered protein tertiary structure. R33Q and L167H mutants retained Ca^2+^ binding capacity but had increased sensitivity to tryptic cleavage and lost Ca^2+^-dependent polymerization.

The Casq2^R33Q^ and Casq2^L167H^ mutants form large oligomers insensitive to Ca^2+^, indicating that they lost Ca^2+^ depend polymerization, and are not able to depolymerize in response to Ca^2+^ depletion, a critical function that affects the RyR2 channel gating response to depletion of Ca^2+^ during muscle contraction^54^. Amazingly, Casq2^D307H^ substitution from aspartic acid to histidine, in the highly conserved hydrophobic core of third thioredoxin-like domain of Casq2, results in the loss of low affinity Ca^2+^ binding to Casq2. This is likely due to disruption of the third thioredoxin-like domain, a highly conserved region in Casq2. Surprisingly, Casq2^D307H^ polymerized in a Ca^2+^-dependent manner, and, just like the wild-type Casq2, it exhibited Ca^2+^-dependent confirmation changes^29,31^. Nevertheless, the loss of low affinity and high capacity Ca^2+^ binding sites, due to severe Casq2^D307H^ misfolding, results in reduced Ca^2+^ storage at the junctional SR, and impaired Casq2/Ca^2+^-dependent regulation of RyR2 activity^51,65^. Animal studies revealed that in Casq2 ^D307H /D307H^ knock-in mice there is a reduction in the abundance Casq2 D307H protein^29,66^. Others reported no effect of D307H mutation of protein stability in either animal model or in cultured cardiomyocytes^31,33,51,65^. Expression of Casq2 L167H mutant did not affect the level of the endogenous wild-type Casq2^34^. However, in R33Q knock-in mice there was a significant reduction in the abundance of Casq2^R33Q/R33Q^ protein^30^. This likely contributes to the CPVT phenotype.

K180R, P329S, G332R, and D351G mutants have not been previously studied with respect to their biochemical properties. K180R is a newly identified Casq2 mutant, and the first autosomal dominant mutant found of Casq2^27^. Knollman’s group recently reported a CPVT-like phenotype in a K180R heterozygous knock-in mouse model^46^. Here we discovered that the Casq2^K180R^ protein has indistinguishable biochemical properties from the wild-type Casq2, including Ca^2+^ binding affinity, secondary structure and conformation change in response to increased Ca^2+^ concentration, protein flexibility and conformation stability upon trypsin proteolysis, protein folding, and Ca^2+^ dependent polymerization. Recently, crystal structure studies of the Casq2^K180R^ maps the mutation to the filament-forming interface^67^, and it was proposed that disrupted Casq2 polymer formation may be responsible for Casq2 mutant-associated CPVT. Casq2 binds directly to the luminal domain of ER stress sensor IRE1α at the junctional SR to prevent the activation of IRE1α^55^. Interestingly, of all mutants tested in this study, only Casq2^K180R^ showed altered binding to the luminal domain of IRE1α. Whether this is associated with CPVT remains to be established.

Casq2^D351G^^39,42^, Casq2^P329S^^39^ and Casq2^G332R^^39^ are three novel Casq2 variants^39,42^ located in a highly conserved third thioredoxin-like domain. Casq2^P329S^ and Casq2^G332R^ have been identified as heterozygous carrier^39^. They are localized in the hydrophobic core of the highly conserved beta-sheet of the third thioredoxin-like domain and are highly conserved throughout the metazoan including Casq1 and pre-duplicate Casq. To our knowledge, there have been no reports on the biochemical and biophysical analysis of these mutants. Casq2^D351G^ showed protein folding and function similar to wild-type protein, whereas Casq2^P329S^ and Casq2^G332R^ showed similar properties to Casq2^D307H^, including severe disruption in protein folding and impaired Ca^2+^ binding, indicative of an important structural and functional role for the highly conserved beta-sheet in the third thioredoxin-like domain of Casq2.

Overall, the three CPVT disease causing Casq2 mutants (R33Q, L167H, D307H) and two heterozygous variants (P329S and G332R) may lead to CPVT via different mechanisms. The third thioredoxin-like fold domain contains one highly conserved beta-sheet consist of four beta-strands, which are essential for correct folding and Ca^2+^ binding, mutations in this region including D307H, P329S, and G332R, and all result in severely misfolded protein with reduced or lost Ca^2+^ binding. R33Q, L167H and K180R are located at the Casq2 front-to-front and back-to-back polymerization interface, causing dysfunction in the protein’s Ca^2+^ depend polymerization/depolymerization that would affect filament formation, as proposed by Titus et al.^67^. This may be the unifying feature of Casq2 mutants association with CPVT^67^.

## Experimental procedures

### Genome databases

The genomes used in the comparative genomics and phylogenetics analyses are publicly available and include the following from NCBI: *Homo sapiens, Canis lupus familiaris, Bos taurus, Oryctolagus cuniculus, Sus scrofa, Rattus norvegicus, Mus musculus, Xenopus tropicalis, Gallus gallus, Taeniopygia guttata, Crocodylus porosus, Latimeria chalumnae, Danio rerio, Callorhinchus milii, Leucoraja erinacea, Branchiostoma floridae, Ciona intestinalis, Helobdella robusta, Drosophila melanogaster, Apis mellifera, Manduca sexta, Daphnia pulex, Parhyale hawaiensis, Parasteatoda tepidariorum, Caenorhabditis elegans, Nematostella vectensis, Mnemiopsis leidyi, Trichoplax adhaerans, Amphimedon queenslandica, Monosiga brevicollis, Salpingoeca rosetta, Capsaspora owczarzaki.* Ensembl: *Helobdella robusta.* Skatebase.org: *Leucoraja erinacea.* hymenopteragenome.org: *Apis mellifera.* wfleabase.org: *Daphnia pulex.*

### Comparative genomics, phylogenetic and sequence alignments

Using *H. sapiens CASQ1* and *CASQ2* sequences as queries, BLASTp (Basic Local Alignment Search Tool protein) searches were performed on the genomes of 28 metazoan organisms (Supplementary Table S1). Reciprocal BLAST was performed to verify the homology of significant hits obtained via forward BLAST. Predictions regarding the homology of a sequence were based on both the E-value and identity score. Hits that displayed the lowest E-value and greatest identity score in both the forward and reciprocal BLAST were predicted as being homologous. In cases of multiple homologous hits, the hit with the greatest identity score was predicted as being potentially orthologous. When no significant hits could be obtained using a BLASTp search, tBLASTn was used to search inside of the genome scaffolds. Additionally, HMMer was also used to search for sequences without significant BLASTp hits. Any potential HMMer hits were then verified using reciprocal BLAST.

For phylogenetic analyses, we used both RAxML consensus trees using 100 bootstraps and MrBayes Bayesian analysis trees with 10 million iterations achieving an average standard deviation of splits frequencies value of less than 0.01. Default parameters on CIPRES for RAxML-HPC v0.8 and MrBayes v3.2.6 on XSEDE were used with the following change for the RAxML trees: an LG4X protein matrix was used with the PROTGAMMA protein substitution model. The clades generated by the RAxML consensus trees were considered significant with node values of at least 50. MrBayes tree clades were considered significant with node probability values of at least 0.8. RAxML consensus trees were generated using Consensus v3.695, while the graphical representation of the phylogenetic results was generated using Figtree v1.3.1. The calsequestrin sequences found using comparative genomics were aligned using MUSCLE v3.8.31 and visualized using MESQUITE v3.2.

### Site-directed mutagenesis

A pET22b *E. coli* expression vector containing full-length recombinant canine *casq2* cDNA with a C-terminus 6xHis tag were used as template to obtain Casq2 mutants. Platinum pfx DNA polymerase (Invitrogen, 11708) was used for site-directed mutagenesis PCR with primers (Supp. Table S2). Methylated non-mutated template plasmids were digested with DpnI, and the correct mutations were confirmed by DNA sequencing. Seven CPVT-related Casq2 mutants (R33Q, L167H, K180R, D307H, P329S, G332R, D351G) were generated.

### Protein purification

cDNA encoding wild-type canine cardiac *casq2* and *casq2* mutants was cloned into pET22b expression vector. Proteins were expressed in *E. coli* BL21(DE3) and purified. Cells were grown in LB medium until the A_600_ reaches 0.6 at 37 °C, then induced with 1 mM IPTG at 37 °C for 3 h. Cells were crashed into a buffer containing 50 mM Tris–HCl, pH 8.0, 300 mM NaCl, and 10% glycerol, then purified by using a HisTrap HP purification column (GE lifesciences, 17524701) and AKTA pure chromatography system (GE lifesciences 29018224). Purification was performed using binding buffer containing 50 mM Tris–HCl, pH 8.0, 300 mM NaCl, and protein eluted with buffer containing 50 mM Tris–HCl, pH 8.0, 300 mM NaCl, and 250 mM imidazole. The 6xHis tagged ER luminal domain of IRE1α (IRE1-NLD) was expressed in COS-1 cells and purified by Ni-NTA agarose chromatography^55^. Protein concentration was determined using Bio-Rad *DC* protein assay (Bio-Rad, 5000111) as recommended by the manufacturer. Protein concentration was also determined by measuring A280 absorbance using Nanovue plus spectrophotometer (GE Healthcare).

### CD analysis

CD spectra were recorded on a Jasco model J-810 spectropolarimeter. Far UV CD spectra were collected with 4.82 μM protein in buffer containing 10 mM NaH_2_PO_4_, pH 7.4, and 5 mM KCl, as NaCl interferes with CD analysis. CD scans were recorded using a quartz cell with a path length of 1 mm, response time of 2 s, scan speed of 10 nm/min, and band width of 1.0 nm. Ca^2+^-induced changes in CD spectra were monitored in the presence of 1 mM of EGTA and 6 mM of CaCl_2_. CD spectra analysis was carried out at 24 °C. The final spectra was an average of 5 measurements, after baseline subtraction. Analysis of the spectra was performed using K2D3^68^. The following calculations were performed for analysis:

Mean residue ellipticity was calculated with formula:

$$\left[\theta \right]={ \theta }_{obs}\times \frac{MRW}{(10\times l\times c)}$$

where [θ] with unit of Deg cm^2^dmol^−1^, θ_obs_ is the observed ellipticity in degrees, $l$ is the optical path-length in cm, $c$ is the protein concentration in g/ml, MRW is the mean residue molecular mass calculated with formula:

$$MRW= \frac{M}{(N-1)}$$

where $M$ is a molecular mass of polypeptide chain in Da, and $N$ is the number of amino acid in the chain.

### MST and thermal denaturation analyses

#### Labelled MST

MST analyses were carried out using a Monolith NT.115 instrument (Nano Temper Technologies, Germany) or Monolith NT.LabelFree instrument (Nano Temper Technologies, Germany). The ER-luminal domain of IRE1α (IRE1-NLD) was labelled using the Monolith NT Protein Labeling Kit RED-NHS (Nano Temper Technologies, cat# MO-C030) following the manufacture’s protocol. All experiments were carried out at room temperature in standard capillaries with 20% LED power (fluorescence lamp intensity) and 40% MST power (IR-laser intensity). The assay buffer contained 50 mM HEPES, pH 7.4, 150 mM KCl, 500 μM CaCl_2_, 250 μM EGTA, 0.05% Tween-20, and 2.5% glycerol. CaCl_2_ and EGTA concentrations were adjusted to obtain the desired free Ca^2+^ concentration: no Ca^2+^ (500 μM EGTA, 500 μM CaCl_2_), 5 mM (500 µM EGTA, 5.5 mM CaCl_2_). Free Ca^2+^ concentration was calculated using the Ca^2+^-EGTA Calculator TS v1.3 web tool^69^.

#### Label-free MST

Ca^2+^ binding to wild-type Casq2 or Casq2 mutants were carried out using a Monolith NT.LabelFree instrument in standard capillaries with 20% LED power and 60% MST power. The proteins were incubated for 10 min in a buffer containing 50 mM HEPES, pH 7.4, 150 mM KCl, 0.1% pluronic F-127, and 50 μM EGTA. An increasing concentration of CaCl_2_ (0.01–20 mM, in 50 mM HEPES, pH 7.4, 150 mM KCl) was used. All MST data was analyzed by Monolith Affinity Analysis v2.2.6 software.

#### Tycho NT.6

Thermal denaturation analysis of wild-type Casq2 or Casq2 mutants was carried out using Tycho NT.6. This label-free system is based on measurement of a protein’s intrinsic tryptophan fluorescence and records a protein’s unfolding profile in real-time as temperature is increased from 35 to 95 °C. Ten µl of 0.25 mg/ml protein was used in buffer containing 50 mM HEPES, pH 7.4, 150 mM KCl, 500 μM CaCl_2_, 250 μM EGTA, 0.05% Tween-20, and 2.5% glycerol.

### Native polyacrylamide gel electrophoresis

To determining the oligomerization state of Casq2 or Casq2 mutants in the presence of the same free Ca^2+^ concentration (167 µM), a discontinuous Tris–glycine polyacrylamide gel system consisting of a 5% stacking gel and a 10% separation gel was used under non-denaturing conditions. Proteins were diluted 3 × with non-denaturing loading dye (240 mM Tris–HCl, pH 6.8, 30% glycerol, and 0.03% bromophenol blue). Proteins were separated in a Mini-PROTEAN electrophoresis chamber (BioRad) in a running buffer containing 25 mM Tris, pH 8.8, and 192 mM glycine, at 100 V, for 2 h at 4 °C. The proteins were stained with Stains-all solution^70^, Coomassie-blue, or transferred to nitrocellulose membrane for immunoblotting analysis.

### Limited proteolysis

Cardiac calsequestrin (Casq2) and mutant proteins were subjected to proteolysis in a buffer containing 50 mM HEPES, pH 7.4, 150 mM KCl, 500 μM CaCl_2_, 250 μM EGTA, 0.05% Tween-20, and 2.5% glycerol. CaCl_2_ and EGTA concentrations were adjusted to the desired free Ca^2+^ concentration: no Ca^2+^ (250 μM EGTA, 250 μM CaCl_2_), 5 mM (250 µM EGTA, 5.25 mM CaCl_2_). Free Ca^2+^ concentration was calculated using the Ca-EGTA Calculator TS v1.3 web tool^69^. Proteins (150 μg of protein in 200 μl) were incubated in a reaction buffer with the desired free Ca^2+^ concentration for 20 min at 25 °C before addition of trypsin at the trypsin/protein ration of 1:50 (w/w), and samples were taken for SDS-PAGE analysis at 1, 2, 5, 10, 20, 30, 60, and 120 min. The samples were mixed with 4 × SDS-PAGE sample buffer (Bio-Rad) containing PMSF, and boiled at 100 °C for 2 min before SDS-PAGE. All experiments were repeated 3 times with protein from 2 separate purifications. The gels were stained with Coomassie Brilliant Blue R-250 (Bio-Rad).

### Cross-linking

The homobiofunctional protein cross linker disuccinimidyl suberate (DSS) (Thermo Scientific Pierce, cat#:21555) was dissolved in DMSO at a final concentration of 10 mM^55^. Protein concentration of wild-type and Casq2 mutant proteins was determined using Bio-Rad *DC* protein assay and the proteins were diluted to a final concentration of 10 µM in a reaction buffer containing 50 mM HEPES, pH 7.4, 150 mM NaCl, 250 μM EGTA, 500 μM CaCl_2_, and 0.05% Tween-20. Proteins were incubated with 20-fold molar excess of DSS for 1 h at 22.5 °C. The reaction was then quenched for 15 min with 100 mM Tris pH 7.4 followed by SDS-PAGE (10% acrylamide). Proteins were transferred to nitrocellulose membrane follow by immunoblotting with mouse anti-6xHis antibodies (ThermoFisher, MA1-21315) or anti-calsequestrin antibodies (abcam, 3516).

### Statistical analysis

Statistical analysis was performed using GraphPad Prism version 7.0. The Student’s t-test was used to compare the mean of two independent groups, and one-way Anova was used to compare the mean of three or more independent groups, with a *p*-value determined to be significant if less than 0.05.

## Supplementary information

Supplementary Information 1.

Supplementary Table S1.
