# Supplementary material for: Phylogenetic and biochemical analysis of calsequestrin structure and association of its variants with cardiac disorders
Source: Sci Rep. 2020 Oct 22;10:18115. doi: 10.1038/s41598-020-75097-3 (PMC7582152; doi:10.1038/s41598-020-75097-3)
Supplement: Supplementary file 1 — Supplementary Information 1. [file 41598_2020_75097_MOESM1_ESM.pdf]

SUPPLEMENTAL INFORMATION

Phylogenetic and biochemical analysis of calsequestrin structure and association of its variants with cardiac disorders.

Qian Wang, Tautvydas Paskevicius, Alexander Filbert, Wenying Qin, Hyeong Jin Kim, Xing-Zhen Chen, Jingfeng Tang, Joel Dacks, Luis B. Agellon, and Marek Michalak

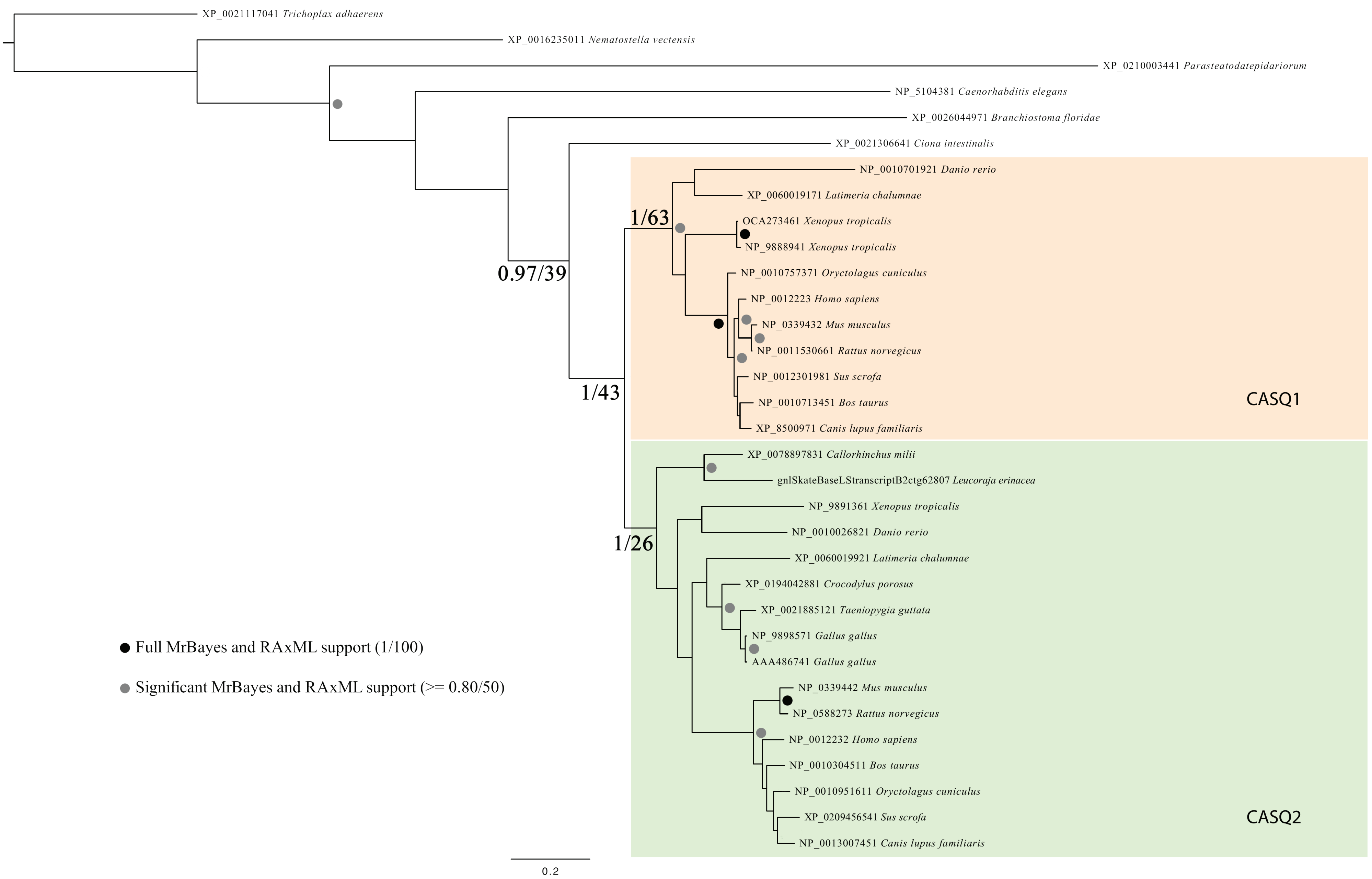

Supplemental Figure S1. Phyolgenetic tree of calsequestrin. A phylogenetic tree was generated by combining MrBayes and RAxML consensus data with Branchiasotma floridae as an outgroup. The Danio rerio casq1 sequence was removed from the dataset used to generate the tree due to long branch attraction causing poor RAxML consensus bootstrap values in previous iterations. The presence of casq1 and casq2 is highlighted by different background colors.

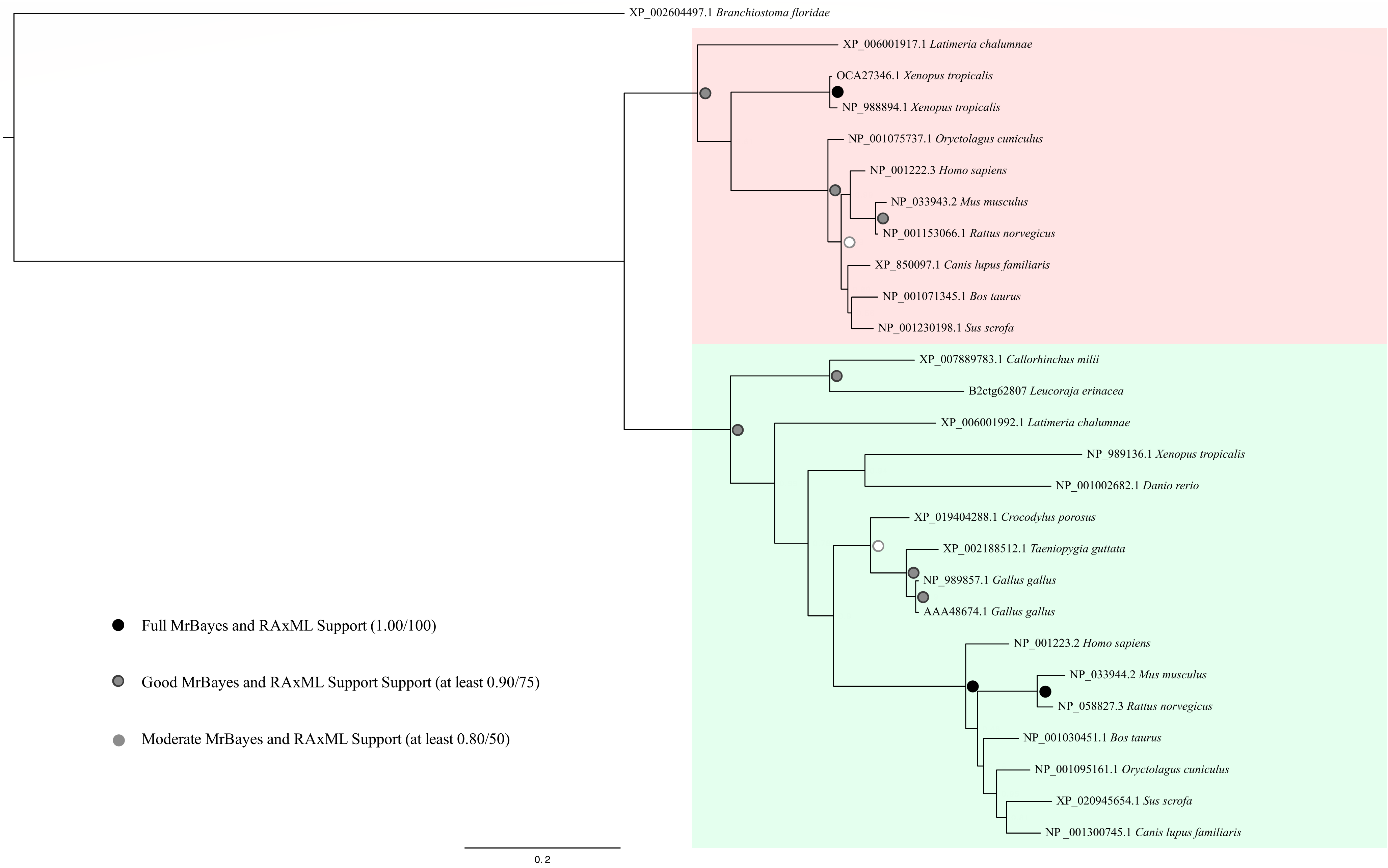

Supplemental Figure S2. Short-branch phyolgenetic tree of calsequestrin. A phylogenetic tree was generated by combining MrBayes and RAxML consensus data as described in Supplementary Figure S1. The presence of casq1 and casq2 is highlighted by different background colors.

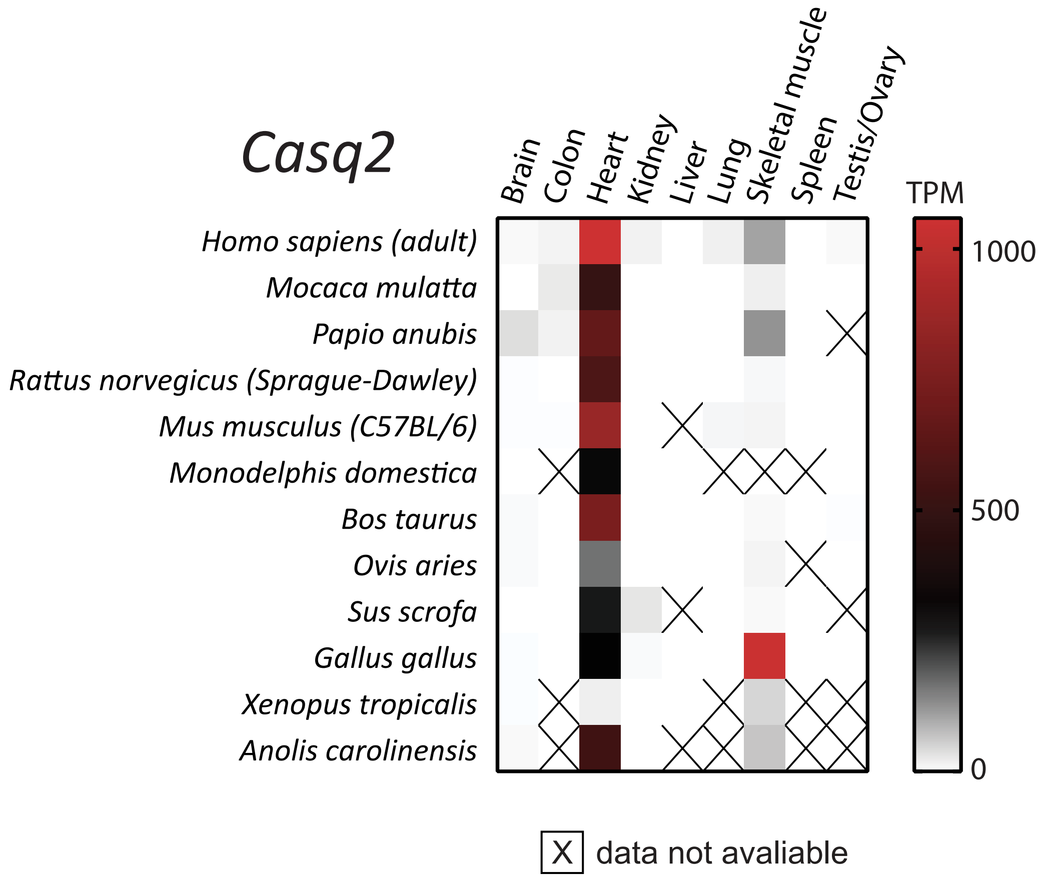

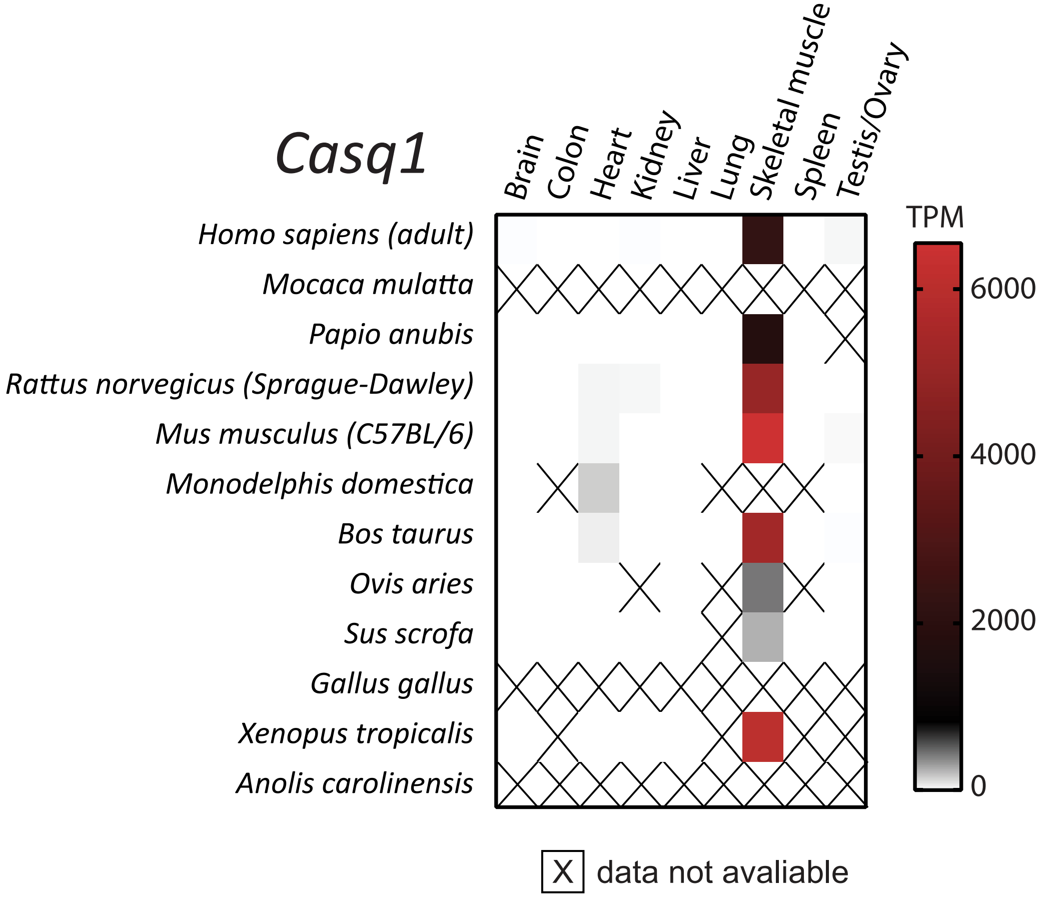

Supplementary Figure S3. Abundance of Casq2 and Casq1 mRNA in selected tissues across species. Data obtained from Expression Atlas (https://www.ebi.ac.uk/gxa/home). TPM, transcripts per million.

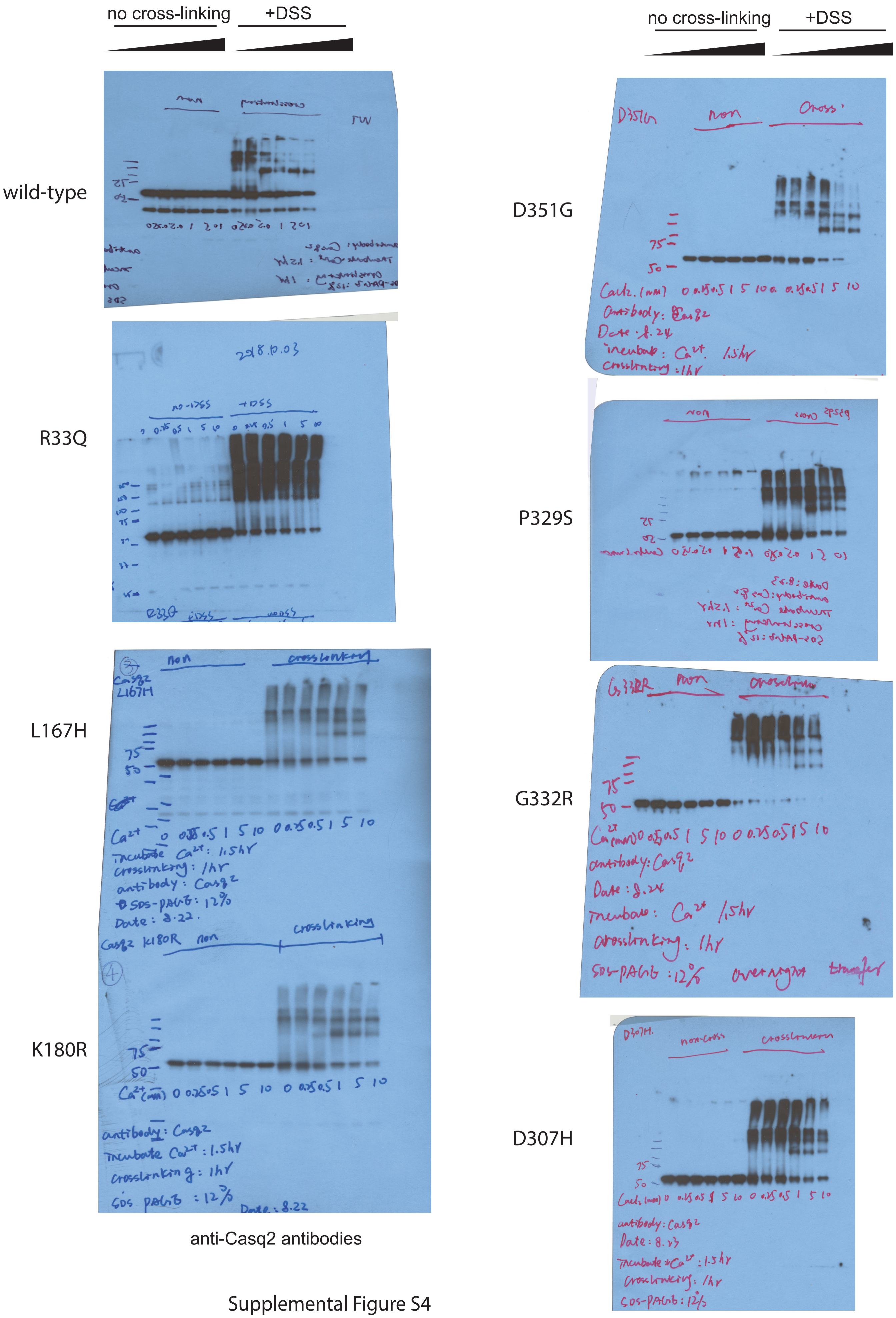

Supplemental Figure S4
The full-length blots for Figure 7B

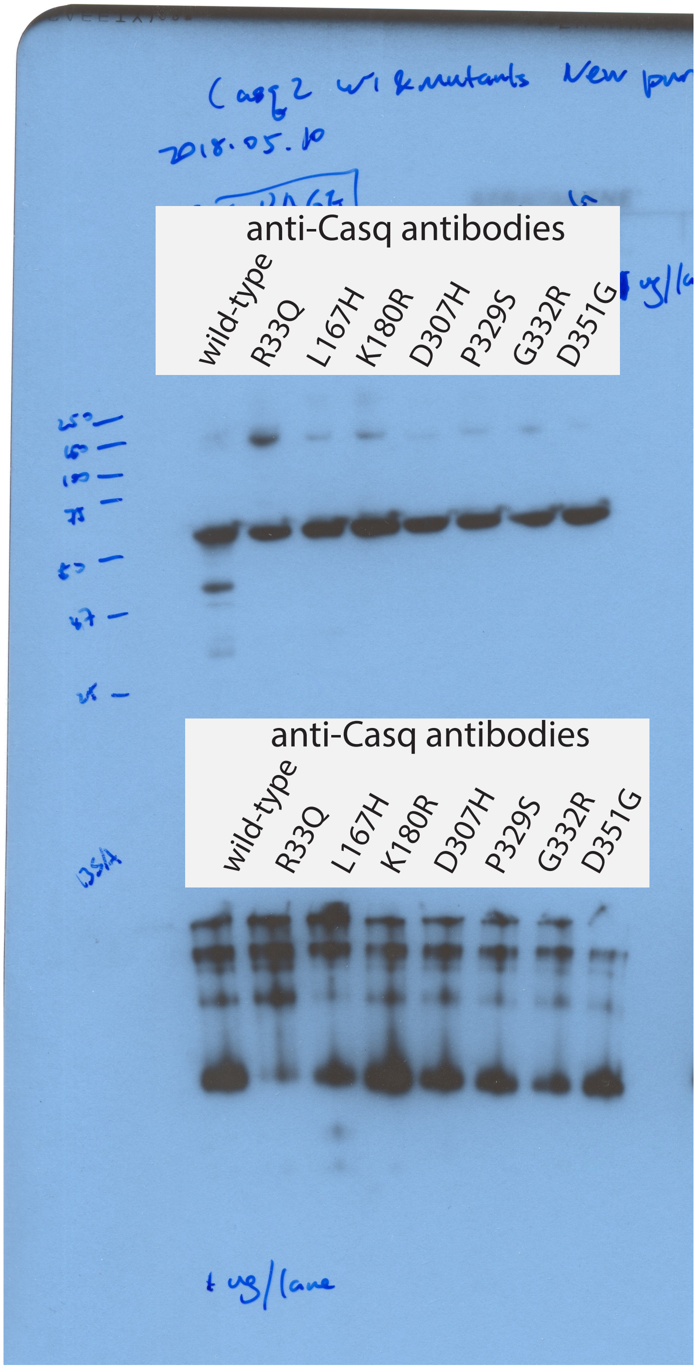

Supplemental Figure S5. The full-length blots for Figure 8

Supplemental Table S1

Query database	Forward query name	Forward query accession number	Subject database	Forward BLAST hit name	Forward BLAST hit accession numbers	Forward BLAST e-values	Reverse BLAST hit names	Reverse BLAST hit accession numbers	Reverse BLAST e-values	Notes
Homo sapiens	calsequestrin-1 preci	NP_001222.3	*Rattus norvegicus*	calsequestrin-1 precursor [Rattus	NP_001153066.1	0	calsequestrin-1 p	0	NP_0012	CASQ1
Homo sapiens	calsequestrin-1 preci	NP_001222.3	*Gallus gallus*	calsequestrin-2 precursor [Gallus	NP_989857.1	0	calsequestrin-2 p	NP_001223	0	Reverse BLAST has identity score of 81 and 91% query cover so this is probably CASQ2
Homo sapiens	calsequestrin-1 preci	NP_001222.3	*Gallus gallus*	calsequestrin-2 precursor [Gallus	NP_989857.1	0	calsequestrin-1 p	NP_001222	0	Reverse BLAST has identity score of 71 and 88% query cover compared to above
Homo sapiens	calsequestrin-1 preci	NP_001222.3	*Gallus gallus*	calsequestrin [Gallus gallus]	AAA48674.1	0	calsequestrin-2 p	NP_001223	0	Reverse BLAST has identity score of 83 and 91% query cover so this is probably CASQ2
Homo sapiens	calsequestrin-1 preci	NP_001222.3	*Gallus gallus*	calsequestrin [Gallus gallus]	AAA48674.1	0	calsequestrin-1 p	NP_001222	0	Reverse BLAST has identity score of 71 and 91% query cover compared to above
Homo	calsequestrin-1 preci	NP_001222.3	*Xenopus*	hypothetical protein XENTR_v90	OCA27346.1	0	calsequestrin-1 p	NP_001222	0	CASQ1
Homo	calsequestrin-1 preci	NP_001222.3	*Xenopus*	calsequestrin-2 precursor [Xenop	NP_989136.1	########	calsequestrin-2 p	NP_001223	########	CASQ2
Homo	calsequestrin-1 preci	NP_001222.3	*Xenopus*	calsequestrin-1 precursor [Xenop	NP_988894.1	########	calsequestrin-1 p	NP_001222	########	CASQ1
Homo	calsequestrin-1 preci	NP_001222.3	*Latimeria*	PREDICTED: calsequestrin-1 [La	XP_006001917.1	0	calsequestrin-1 p	NP_001222	0	CASQ1
Homo	calsequestrin-1 preci	NP_001222.3	*Latimeria*	PREDICTED: calsequestrin-2 [La	XP_006001992.1	0	calsequestrin-2 p	NP_001223	0	CASQ2
Homo	calsequestrin-1 preci	NP_001222.3	*Danio rerio*	calsequestrin-2 precursor [Danio	NP_001002682.1	########	calsequestrin-2 p	NP_001223	0	CASQ2
Homo	calsequestrin-1 preci	NP_001222.3	*Danio rerio*	calsequestrin-1 precursor [Danio	NP_001070192.1	########	unnamed protein	BAC86117.1	0	CASQ1?
Homo	calsequestrin-1 preci	NP_001222.3	*Danio rerio*	calsequestrin-1 precursor [Danio	NP_001070192.1	########	calsequestrin-1 p	NP_001222	########	CASQ1?
Homo	calsequestrin-1 preci	NP_001222.3	*Callorhinchus*	PREDICTED: calsequestrin-2-lik	XP_007889783.1	########	calsequestrin-2 p	NP_001223	0	Only one forward
Homo	calsequestrin-1 preci	NP_001222.3	*Callorhinchus*	PREDICTED: calsequestrin-2-lik	XP_007889783.1	########	calsequestrin-1 p	NP_001222	0	tBLASTn did not
Homo	calsequestrin-1 preci	NP_001222.3	*Leucoraja*	gnl|SkateBase|LS-transcriptB2-c	ctg62807	########	calsequestrin-2 p	NP_001223	0	No hits on NCBI,
Homo	calsequestrin-1 preci	NP_001222.3	*Petromyzon*							No hits on NCBI,
Homo	calsequestrin-1 preci	NP_001222.3	*Branchiostoma*	hypothetical protein BRAFLDRA	XP_002604497.1	7.00E-45	Chain A, Ca2+ C	3UOM_A	8.00E-57	Appears to be

Homo	calsequestrin-1 preci	NP_001222.3	*Branchiostoma*	hypothetical protein BRAFLDRA	XP_002604497.1	7.00E-45	calsequestrin-1 p	NP_001222	1.00E-56	
Homo	calsequestrin-1 preci	NP_001222.3	*Ciona intestinalis*	PREDICTED: calsequestrin-2 [C	XP_002130664.1	########	Chain A, Human	5CRE_A	########	CASQ present but
Homo	calsequestrin-1 preci	NP_001222.3	*Ciona intestinalis*	PREDICTED: calsequestrin-2 [C	XP_002130664.1	########	calsequestrin-1 p	NP_001222	########	
Homo	calsequestrin-1 preci	NP_001222.3	*Helobdella robusta*							No hits on NCBI or
Homo	calsequestrin-1 preci	NP_001222.3	*Drosophila melanogaster*							No hits on NCBI
Homo	calsequestrin-1 preci	NP_001222.3	*Caenorhabditis eleg*	Calsequestrin [Caenorhabditis el	NP_510438.1	2.00E-53	Chain A, Human	5CRE_A	3.00E-51	CASQ present
Homo	calsequestrin-1 preci	NP_001222.3	*Caenorhabditis eleg*	Calsequestrin [Caenorhabditis el	NP_510438.1	2.00E-53	calsequestrin-1 p	NP_001222	5.00E-51	
Homo	calsequestrin-1 preci	NP_001222.3	*Nematostella vecte*	predicted protein [Nematostella v	XP_001623501.1	9.00E-55	Chain A, Human	5CRH_A	9.00E-54	
Homo	calsequestrin-1 preci	NP_001222.3	*Mnemiopsis leidyi*							No hits on NCBI
Homo	calsequestrin-1 preci	NP_001222.3	*Trichoplax adhaere*	hypothetical protein TRIADDRAF	XP_002111704.1	6.00E-48	calsequestrin-2 p	NP_001223	2.00E-50	
Homo	calsequestrin-1 preci	NP_001222.3	*Amphimedon queenslandica*							No hits on NCBI
Homo	calsequestrin-1 preci	NP_001222.3	*Monosiga brevicollis*							Only 1 hit with e-
Homo	calsequestrin-1 preci	NP_001222.3	*Salpingoeca rosetta*	hypothetical protein PTSG_0215	XP_004996635.1	5.00E-09	protein disulfide-	NP_000909	3.00E-86	Not CASQ
Homo	calsequestrin-1 preci	NP_001222.3	*Capsaspora owczarzaki*							Not hits on NCBI
Homo	calsequestrin-1 preci	NP_001222.3	*Oryctolagus cunicul*	calsequestrin-1 precursor [Oryct	NP_001075737.1	0.00E+00	calsequestrin-1 p	NP_001222	0.00E+00	CASQ1
Homo	calsequestrin-1 preci	NP_001222.3	*Mus musculus*	calsequestrin-1 precursor [Mus r	NP_033943.2	0.00E+00	calsequestrin-1 p	NP_001222	0.00E+00	CASQ1
Homo	calsequestrin-1 preci	NP_001222.3	*Apis mellifera*		XM_001121993.4	6.52E-05				No hits on NCBI,
Homo	calsequestrin-1 preci	NP_001222.3	*Manduca sexta*							No hits on NCBI
Homo	calsequestrin-1 preci	NP_001222.3	*Canis lupus familiar*	calsequestrin-1 [Canis lupus fam	XP_850097.1	0	calsequestrin-1 p	NP_001222	0	CASQ1
Homo	calsequestrin-1 preci	NP_001222.3	*Sus scrofa*	calsequestrin-1 precursor [Sus s	NP_001230198.1	0	calsequestrin-1 p	NP_001222	0	CASQ1
Homo	calsequestrin-1 preci	NP_001222.3	*Bos taurus*	calsequestrin-1 precursor [Bos ta	NP_001071345.1	0	calsequestrin-1 p	NP_001222	0	CASQ1
Homo	calsequestrin-1 preci	NP_001222.3	*Crocodylus porosus*	PREDICTED: calsequestrin-2 [C	XP_019404288.1	0	calsequestrin-2 p	NP_001223.	0	CASQ2?
Homo	calsequestrin-1 preci	NP_001222.3	*Taeniopygia guttata*	PREDICTED: calsequestrin-2 [T	XP_002188512.1	0	calsequestrin-2 p	NP_001223	0	CASQ2?
Homo	calsequestrin-1 preci	NP_001222.3	*Parasteatoda tepida*	calsequestrin-2-like [Parasteatoc	XP_021000344.1	4.00E-21	unnamed protein	BAG58422.1	2.00E-23	Appears to be
Homo	calsequestrin-1 preci	NP_001222.3	*Parasteatoda tepida*	calsequestrin-2-like [Parasteatoc	XP_021000344.1	4.00E-21	calsequestrin-2 p	NP_001223	4.00E-22	Appears to be
Homo	calsequestrin-1 preci	NP_001222.3	*Daphnia pulex*							No sig. hits on
Homo	calsequestrin-1 preci	NP_001222.3	*Parhyale hawaiensis*							No sig. hits on

Supplementary Table S2

DNA primers used to generate cDNA encoding calsequestrin mutants.

	Forward primer (5' to 3')	Reverse primer (5' to 3')
R33Q	GATGGCAAAGACCAGGTGGTCAGT CTCACTG	CAGTGAGACTGACCACCTGGTCTTTGC CATC
L167H	GAGGACCAGATCAAACACATTGGC TTTTTCAAG	CTTGAAAAAGCCAATGTGTTTGATCTG GTCCTC
K180R	GTCAGAGTATTATAGGGCTTTTGAG GAGGC	GCCTCCTCAAAAGCCCTATAATACTCT GAC
D307H	GCATCGTGTGGATTCACCCGGATG ACTTTC	GAAAGTCATCCGGGTGAATCCACACG ATGC
P329S	TTGACCTATTCAAGTCACAGATCGG GGTGGT	ACCACCCCGATCTGTGACTTGAATAGG TCAA
G332R	TCAAGCCACAGATCCGTGTGGTGA ATGTGAC	GTCACATTCACCACACGGATCTGTGGC TTGA
D351G	TTCCTGATGATGATGGCCTGCCCAC AGCTGA	TCAGCTGTGGGCAGGCCATCATCATCA GGAA

Mutated residues are shown in red
